# Supplementary material for: Free ISG15 Inhibits the Replication of Peste des Petits Ruminants Virus by Breaking the Interaction of Nucleoprotein and Phosphoprotein
Source: Microbiol Spectr. 2022 Aug 29;10(5):e01031-22. doi: 10.1128/spectrum.01031-22 (PMC9603952; doi:10.1128/spectrum.01031-22)
Supplement: Supplemental file 1 — Fig. S1. Download spectrum.01031-22-s0001.pdf, PDF file, 0.2 MB [file spectrum.01031-22-s0001.pdf]

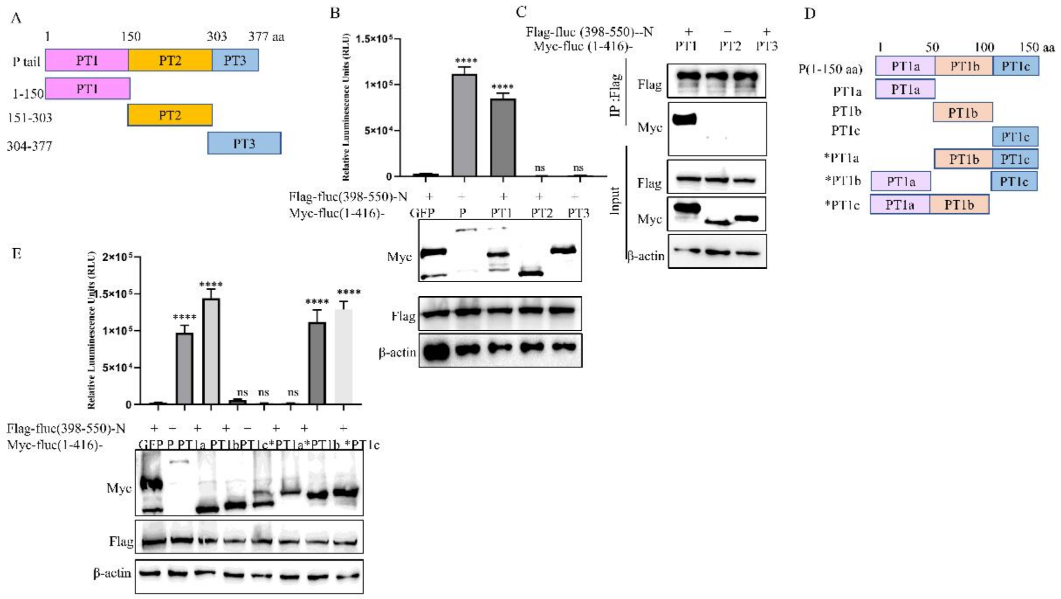

Fig. S1 N and ISG15 proteins interact with P aa 1 to 50, respectively. (A) Schematic representation of the strategy for constructing truncated P aa 1 to 377. (B) HEK-293T cells were cotransfected with Flag-fluc (398-550)-N, and Myc-fluc (1-416)-GFP, or truncated P, PT1, PT2, PT3. At 24 hpt, cell lysates were collected to detect luciferase intensity and detected using western blotting analysis with anti-Flag, anti-Myc, and anti-β-actin. (C) HEK-293T cells were cotransfected with Flag-fluc (398-550)-N, and Myc-fluc (1-416)-GFP, or truncated P, PT1, PT2, PT3. At 24 hpt, cells were harvested and immunoprecipitated with anti-Flag antibody. Immunoblot analysis was performed with anti-Myc and anti-Flag antibodies. Expression levels of the proteins were analyzed by immunoblot analysis of the lysates with anti-Flag, anti-Myc, and anti-β-actin antibodies. (D) Schematic representation of the strategy for constructing truncated P aa 1 to 150. (E) HEK-293T cells were cotransfected with Flag-fluc (398-550)-N, and Myc-fluc (1-416)-GFP, or truncated P, PTa, PT1b, PT1c, *PTa, *PT1b, *PT1c. At 24 hpt, cell lysates were collected to detect luciferase intensity and detected using western blotting analysis with anti-Flag, anti-Myc, and anti-β-actin.
